# Fascitis necrotizante: revisión y reporte de caso

**DOI:** 10.21142/2523-2754-1204-2024-224

**Published:** 2024-11-23

**Authors:** Sebastián Martínez Venegas, Nicolás Olivares, María José Pérez

**Affiliations:** 1 Hospital Dr. Franco Ravera Zunino. Rancagua, Chile. sebamartinezven@outlook.com , mariajoseep@hotmail.com Hospital Dr. Franco Ravera Zunino Rancagua Chile sebamartinezven@outlook.com mariajoseep@hotmail.com; 2 Escuela de Odontología, Pontificia Universidad Católica de Chile. Santiago, Chile. nholivaresv@gmail.com Pontificia Universidad Católica de Chile Escuela de Odontología Pontificia Universidad Católica de Chile Santiago Chile nholivaresv@gmail.com

**Keywords:** fascitis necrotizante, facial, diagnóstico, tratamiento, necrotizing fasciitis, facial, diagnosis, treatment

## Abstract

**Introducción:**

La fascitis necrotizante (FN) en una enfermedad severa y agresiva, de rápida progresión y asociada con enfermedades sistémicas. Su presentación es rara en la región de cabeza y cuello.

**Materiales y método:**

Presentamos el caso de un paciente de 69 años con diabetes mellitus no controlada, que acude a urgencias por un cuadro de 3 días de evolución, caracterizado por dolor molar derecho con posterior aumento de volumen facial derecho y celulitis periorbitaria. El cuadro mostró una evolución cíclica influenciada por las comorbilidades existentes, por lo que el paciente fue sometido a tratamiento tanto quirúrgico como médico por un equipo multidisciplinario. Adicionalmente realizamos una revisión de la literatura sobre la FN.

**Resultados:**

Los resultados de supervivencia del paciente fueron favorables; sin embargo, las secuelas fueron incapacitantes tanto estética como funcionalmente. La revisión de la literatura reveló la rareza de tales casos y las peculiaridades del caso presentado, en comparación con los reportados en la literatura hasta el momento.

**Conclusiones:**

Un diagnóstico temprano a partir de sus manifestaciones clínicas y estudios complementarios puede llevar a un correcto manejo de la enfermedad.

## Abstract (en)

**Introduction:**

Necrotizing Fasciitis (NF) is a severe and aggressive disease with rapid progression associated with systemic diseases, its presentation is rare in the head and neck region.

**Materials and Methods:**

We present the case of a 69-year-old patient who consulted urgently for a 3-day history characterized by right molar pain with subsequent increase in right facial volume and periorbital cellulitis, with uncontrolled diabetes mellitus. The condition showed a cyclical evolution influenced by existing comorbidities, with the patient undergoing both surgical and medical treatment with a multidisciplinary team. Additionally, we carried out a review of the literature on FN.

**Results:**

The patient's survival outcomes were favorable; However, the consequences were disabling both aesthetically and functionally. The literature review revealed the rarity of such cases and the peculiarities of the presented case compared to those reported in the literature so far.

**Conclusions:**

An early diagnosis based on its clinical manifestations and complementary studies can lead to correct management of the disease.

## INTRODUCCIÓN

La fascitis necrotizante (FN) es una infección poco común que avanza rápidamente a través de varios planos anatómicos, incluyendo la piel, el tejido celular subcutáneo y las fascias superficiales y profundas ^1^, con la consiguiente liberación de toxinas bacterianas. Este proceso conduce a la isquemia tisular y la necrosis licuefactiva de los tejidos ^2^. Se caracteriza por una extensa necrosis de estos planos y los tejidos circundantes, lo que genera una grave toxicidad sistémica que puede ser potencialmente mortal ^3^. Es considerada una enfermedad autolimitante, pero con un alto riesgo de morbilidad, y su tasa de mortalidad alcanza un promedio del 32,2%. La incidencia de la FN se estima entre 500 y 1000 casos al año, con una prevalencia mundial de 0,40 casos por cada 100 000 habitantes. Aunque puede afectar a personas de todas las edades, los hombres mayores de 50 años tienen una mayor susceptibilidad, con una relación de 3 a 1, en comparación con las mujeres ^1^. Esta enfermedad es más común en el tronco, las extremidades y el perineo ^3^, mientras que su aparición es rara en la región de la cabeza y el cuello, con solo el 1-10% de los casos ^4^.

Entre los eventos precipitantes de la fascitis necrotizante (FN) se destacan el trauma y las infecciones de origen odontogénico. El trauma se relaciona estrechamente con la aparición de la enfermedad, ya que muchos pacientes tienen antecedentes de traumas menores o mayores, que pueden involucrar lesiones externas y heridas quirúrgicas. Estos factores, combinados con las comorbilidades del paciente, pueden promover el desarrollo de la FN ^5^. La mayoría de los casos de FN cervicofacial son de origen odontogénico. Así, al igual que otras infecciones odontogénicas, el diagnóstico suele basarse en los hallazgos clínicos y radiológicos ^6^, pero la enfermedad puede diagnosticarse fácilmente de manera errónea como un absceso odontogénico común, celulitis o erisipela en sus primeras etapas ^7^. Entre las comorbilidades más frecuentes de los pacientes con FN encontramos la diabetes mellitus, inmunodeficiencias, insuficiencia cardiaca crónica, cirrosis hepática, hipertensión y enfermedad vascular periférica ^2^.

Los pacientes con fascitis necrotizante (FN) muestran síntomas como dolor, edema y eritema, además de cambios fisiológicos como taquicardia, fiebre, hipotensión y taquipnea. Estos síntomas y signos, especialmente cuando se combinan con eritema, son indicativos y útiles para el diagnóstico de FN ^8^. Las manifestaciones cutáneas pueden incluir formación de ampollas, necrosis localizada, crepitación y gases subcutáneos. El diagnóstico de la FN en una etapa temprana puede ser un desafío debido a la ausencia de síntomas específicos. Si no se trata, la FN puede progresar rápidamente a un *shock* séptico y provocar la muerte ^9^. El diagnóstico está basado principalmente en el examen clínico, signos y síntomas, los cuales deben ser complementados con exámenes de laboratorio e imagenológicos. El tratamiento está basado en una combinación de antibióticos intravenosos de amplio espectro, desbridamientos quirúrgicos regulares y, en algunos casos, injertos de piel de espesor parcial o total ^1^^,^^7^^,^^10^.

Presentamos el caso de un paciente de 69 años con diabetes mellitus no controlada, que acude a urgencias por un cuadro con 3 días de evolución, caracterizado por dolor molar derecho con posterior aumento de volumen facial derecho y celulitis periorbitaria. Adicionalmente, se realizó una revisión de la literatura sobre la FN.

## MATERIALES Y MÉTODOS

### Reporte de caso

Paciente masculino de 69 años con antecedentes de hipertensión arterial, diabetes mellitus tipo II y artrosis, acude a urgencias del Hospital Franco Ravero Zunino (HFRZ) el día 27 de marzo de 2024 por un cuadro con 3 días de evolución, caracterizado por dolor molar derecho con posterior aumento de volumen facial derecho y celulitis periorbitaria, ocasionada en contexto de caída de nivel de 4 días de evolución antes de su ingreso. Los registros médicos del paciente relevaron antecedentes de mal control metabólico asociado a cetoacidosis diabética en 2022 y pie diabético. 

**Figura 1:**
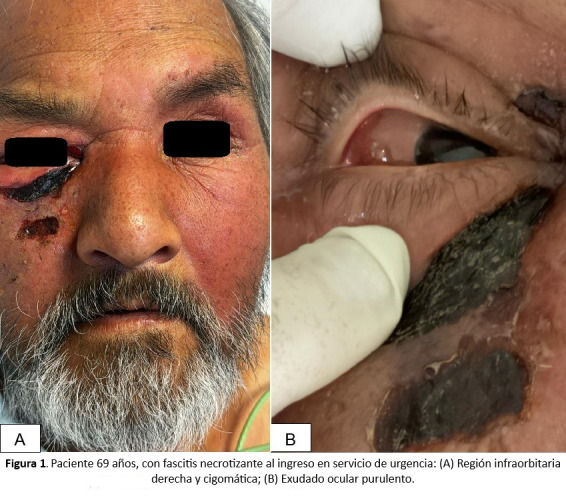
Paciente de 69 años con fascitis necrotizante al ingreso en el servicio de urgencia. A) Región infraorbitaria derecha y cigomática. B) Exudado ocular purulento.

Al examen físico se evidencia dolor exacerbado, edema y eritema en región periorbitaria derecha, asociado con lesiones necróticas en región infraorbitaria derecha vinculado a quemosis, exudado purulento en globo ocular derecho, limitación de apertura ocular y disminución de agudeza visual (Figura 1). 

**Figura 2:**
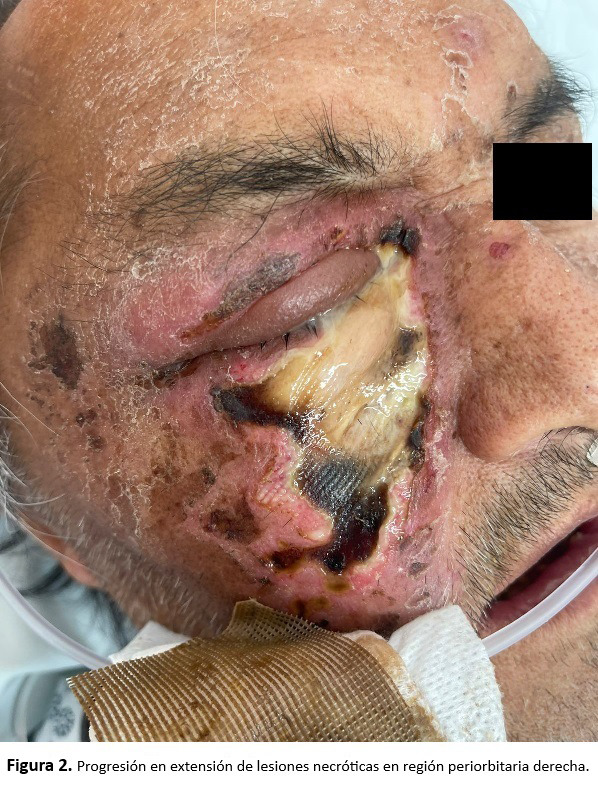
Progresión en extensión de lesiones necróticas en región periorbitaria derecha.

Al examen intraoral se encontró ausencia de piezas dentarias, presencia de múltiples restos radiculares y mal control de higiene. Durante su estadía en urgencias, el paciente fue sometido a una reevaluación, en la que se observó una rápida progresión de las lesiones necróticas, las cuales aumentaron su extensión y comprometieron la región geniana y palpebral derecha (Figura 2).

Los exámenes de laboratorio revelaron leucocitosis con neutrofilia (23 400 mm^3^), procalcitonina (PCT: 10,14), parámetros inflamatorios elevados (PCR: 44,4), hiperglucemia (412 mg/dl) y cetonemia (+++). Los resultados de gases fueron compatibles con una cetoacidosis metabólica asociada a una sepsis de foco cutáneo. Las imágenes en urgencia, realizadas mediante tomografía axial computarizada (TAC) con contraste al ingreso del paciente, confirman la presencia de edema y cambios inflamatorios periorbitarios preseptales en relación con la región cigomática derecha, sin compromiso orbitario postseptal. Dada la presentación clínica del paciente y su condición médica general, se decidió su ingreso a UCI para monitorización y manejo intrahospitalario.

El manejo intrahospitalario se inició con antibioterapia empírica con ampicilina/sulbactam, complementado con cloxacilina y aseo quirúrgico en pabellón central. Bajo anestesia general con intubación orotraqueal, se realizó un abordaje que involucró al equipo de cirugía maxilofacial del hospital. Se realizó un desbridamiento quirúrgico extenso, que comprende piel y tejido celular subcutáneo, lo que implicó la extirpación de todos los tejidos necróticos y desvitalizados. El tejido extirpado fue enviado a biopsia y cultivo. Durante el periodo posoperatorio, el paciente permaneció en UTI y evolucionó adecuadamente, afebril, con disminución de edema facial y resolución de falla renal aguda. 

El análisis microbiológico de las muestras del paciente reveló una infección polimicrobiana, con la identificación de *Staphylococcus aureus*, *Staphylococcus lugdunensis* y *Staphylococcus epidermidis*. Según los resultados microbiológicos, el equipo de infectología recomendó continuar el tratamiento empírico con ampicilina/sulbactam y cloxacilina. Después de cuatro días, se realizó una nueva evaluación por parte de infectología, en la que se sugirió suspender el tratamiento empírico con ampicilina/sulbactam e iniciar una terapia empírica con cotrimoxazol y cloxacilina.

### Revisión sistemática

Se realizó una revisión de la literatura según las recomendaciones indicadas en “Preferred Reporting Items for Systematic Reviews and Meta-Analyses extension for Scoping-reviews” (PRISMA-ScR).

### Fuentes de información y criterios de elegibilidad

Una búsqueda general fue realizada en la base de datos PubMed utilizando los términos de búsqueda “(Necrotizing fasciitis) AND (facial)) AND (diagnosis)”. La estrategia de búsqueda se ideó pensando en la pregunta de investigación: ¿Cuáles son los signos, síntomas, comorbilidades y tratamientos más frecuentes en pacientes diagnosticados con fascitis necrotizante, según la literatura disponible? El principal objetivo de esta revisión fue examinar la literatura disponible sobre la fascitis necrotizante para describir los signos, síntomas, comorbilidades y tratamiento en pacientes afectados por esta condición.

El diseño de los estudios incluidos fue observacional retrospectivo o prospectivo, reporte y serie de casos. Se consideró como límite inferior el año 2014 y como superior, enero de 2024. Los estudios excluidos fueron revisiones narrativas, comentarios, estudios en animales o aquellos cuyos datos no podían ser extraídos de forma fiable, y reportes de caso en pacientes pediátricos.

### Screening y selección de estudios

Dos revisores independientes (SM y NO) fueron encargados de filtrar los documentos por título y *abstract* utilizando los criterios de inclusión previamente mencionados. En caso de existir desacuerdo, un tercer revisor (MP) medió para resolver las discrepancias. Los artículos seleccionados fueron dispuestos a lectura completa por dos revisores (SM y NO), quienes determinaron si debían o no ser incluidos en el análisis final de datos. En caso de existir discrepancias, un tercer revisor (MP) medió y arbitró la decisión final.

## RESULTADOS

**Figura 3:**
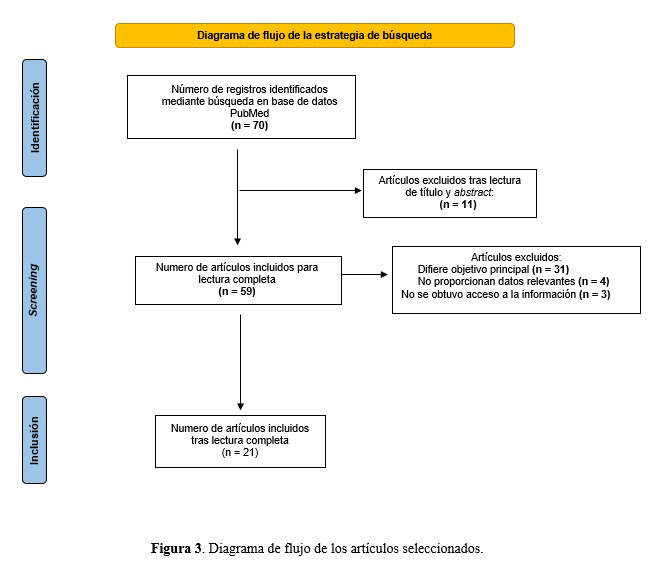
Diagrama de flujo de los artículos seleccionados.

Mediante la estrategia de búsqueda, se identificó un total de 70 referencias, de las cuales, tras la lectura de título y *abstract*, 11 centraron su contenido en reportes de caso en pacientes pediátricos. Por lo tanto, se acordó que 59 artículos serían considerados para su lectura completa. No se pudo obtener acceso a la información en 3 documentos. Además, 31 artículos abordaron aspectos que se desviaron de los objetivos principales de la revisión. Solo 4 artículos no proporcionaron ningún dato relevante. Finalmente, solo 21 documentos fueron incluidos en esta revisión (Figura 3).

Respecto de las características demográficas de los artículos incluidos, se incluyó un total de 21 casos, que corresponden a 11 hombres (52,4%) y 10 mujeres (47,6%). La edad media de los pacientes fue de 46 años. 

Entre los casos observados, en relación con la etiología de la patología, se observó que, en 8 casos (38,0%), el trauma fue mencionado como el factor etiológico principal. Le siguieron las causas de origen odontogénico en 7 casos (33,3%), complicaciones tras procedimientos quirúrgicos en 3 casos (14,3%), otras causas en 2 casos (9,52%) y afecciones dermatológicas en 1 caso (4,76%).

Se documentaron las comorbilidades de los pacientes. Entre el total de artículos, se identificaron 14 casos (66,6%) con comorbilidades asociadas, mientras que en 6 casos (28,6%) no se registraron comorbilidades. Además, en 1 de los casos (4,76%) no fue posible determinar con precisión el historial médico. Las comorbilidades más prevalentes fueron la diabetes mellitus, presente en 6 casos (42,9%), y el consumo crónico de alcohol, también con 6 casos (42,9%). Se observaron enfermedades autoinmunes y genéticas en 3 casos (21,4%), hipertensión arterial en 2 casos (14,3%), y se reportó un caso (7,14%) de consumo crónico de drogas y estupefacientes. Por tanto, se identificaron un total de 10 casos de inmunosupresión.

Los signos y síntomas más comunes dentro de los estudios incluidos fueron dolor, edema, eritema y necrosis tisular de la zona afectada. Además, se observó en algunos de los casos reportados fiebre, hipotensión y secreción purulenta. Solamente en uno de los casos se describió encefalopatía, absceso renal y neumonía asociada con la FN. 

En todos los casos se indicó terapia antibiótico-endovenosa y se realizó desbridamiento quirúrgico como parte del tratamiento. En la mayoría de ellos, se procedió a cubrir el defecto mediante injerto de piel, ya sea de espesor total o parcial, así como mediante colgajos. La enucleación del globo ocular se reportó solo en un caso (Tabla 1). 

**Tabla 1:** Descripción de la evidencia en los estudios evaluados

Autor, Año	Sexo	Edad	Etiología	Comorbilidades	Signos y síntomas	Antibioterapia	Tratamiento
Pertea Mihaela *et al*. 2023 (12)	Masculino	67	Trauma	Diabetes mellitus	Edema hemifacial izquierdo	Ceftriaxona y colistina endovenosa, 3 UI c/24 h	Desbridamiento quirúrgico
				Consumo crónico de alcohol	Necrosis tisular		Enucleación globo ocular
				Trastornos psiquiátricos	Enfisema subcutáneo		Fasciectomía y necrotomía
Nyirjesy *et al*. 2023 (13)	Femenino	58	Lesión de caries	Diabetes mellitus	Dolor	Vancomicina	Desbridamiento quirúrgico
					Edema	Ampicilina/Sulbactam	Injerto piel espesor parcial
						Fluconazol	Colgajo rotado
Nripen *et al*. 2023 (14)	Masculino	30	Extracciones	No presenta	Edema palpebral derecho	Ceftriaxona	Desbridamiento quirúrgico
			Profilaxis dental		Eritema	Amikacina	
			Remoción de caries				
			Endodoncia				
Huang *et al*. 2023 (15)	Masculino	26	Trauma	No presenta	Dolor, edema periorbitario bilateral, hipotensión	Vancomicina/piperacilina	Desbridamiento quirúrgico
						Tazobactam	
Mosenia *et al*. 2022 (16)	Masculino	39	Lesión de caries	Consumo crónico de alcohol	Dolor, edema, eritema región periorbitaria izquierda	Clindamicina	Desbridamiento quirúrgico
						Vancomicina	Etmoidectomía
						Ceftriaxona	Exodoncia dental
						Metronidazol	Antrostomía maxilar
Woon Lee Da *et al*. 2022 (17)	Masculino	43	Embolismo pulmonar	Diabetes mellitus	Dolor, edema y eritema	Cefotaxima	Desbridamiento quirúrgico
					Fiebre y encefalopatía	Ceftriaxona 2 g/día	Descompresión quirúrgica
					Absceso renal y neumonía		
Ling Jin *et al*. 2021 (18)	Masculino	48	Absceso periamigdaliano	Diabetes mellitus	Dolor, disfagia, odinofagia, terciana	Meropenem tigeciclina	Desbridamiento quirúrgico
						Fluconazol	
Edwards *et al*. 2020 (19)	Masculino	33	Extracción dental	Consumo de cocaína y metanfetamina	Dolor, edema periorbitario izquierdo y submandibular	Vancomicina/gentamicina	Desbridamiento quirúrgico, drenaje quirúrgico
						Piperacilina/tazobactam	
						Linezolid	
Aman Negi *et al*. 2020 (20)	Masculino	32	Trauma	No informa	Dolor, edema, eritema, secreción purulenta, fiebre	Penicilina G	Desbridamiento quirúrgico
						Clindamicina	Injerto piel espesor parcial
McCabe *et al*. 2020 (21)	Femenino	56	Trauma	Hipertensión arterial	Edema, eritema periorbitario bilateral	Vancomicina	Desbridamiento quirúrgico
				Consumo crónico de alcohol		Bencilpenicilina	
				Obesidad		Meropenem	Injerto piel espesor total
						Clindamicina	
Ting *et al*. 2020 (22)	Femenino	35	Trauma subgaleal asociado a hematoma	Consumo crónico de alcohol	Dolor, edema periorbitario bilateral, hipotensión	Penicilina	Desbridamiento quirúrgico
						Clindamicina	Injerto piel espesor total
						Cefalexina	
Jongweon *et al*. 2019 (23)	Femenino	79	Trauma	Hipertensión arterial	Edema, eritema hemifacial derecho	Ceftriaxona	Desbridamiento quirúrgico
						Clindamicina	
Haen *et al*. 2019 (24)	Femenino	57	Ritidectomía	No presenta	Edema, eritema, lesiones necróticas, fiebre	No especifica	Desbridamiento quirúrgico
							Orbitotomía bilateral
Steybe *et al*. 2019 (25)	Femenino	77	Extracción dental, osteonecrosis medicamentosa	Diabetes mellitus	Dolor, edema, eritema región submandibular y submentoniana	Penicilina	Desbridamiento quirúrgico
				Osteoporosis		Metronidazol	Resección quirúrgica
						Cefuroxima	
Pang Yun *et al*. 2018 (26)	Masculino	44	Lesión de caries	Diabetes mellitus	Dolor, edema, necrosis, disminución de agudeza visual, fiebre,	Vancomicina imipenem	Desbridamiento quirúrgico
				Mal control de higiene		Cilastatina	
							Injerto piel espesor parcial
Deneubourg *et al*. 2018 (27)	Femenino	30	Trauma	No presenta	Dolor, fiebre, eritema, edema palpebral bilateral, necrosis palpebral izquierda, quemosis	Penicilina G	Desbridamiento quirúrgico
						Piperacilina/tazobactam	Injerto piel espesor total
						Gentamicina	
						Clindamicina	
Tent *et al*. 2018 (28)	Femenino	47	Trauma	Psoriasis	Dolor, edema, eritema región occipital	Colistina UI c/8 h	Desbridamiento quirúrgico
				Consumo crónico de alcohol		Vancomicina 1000 g	
				Consumo de tabaco		Imipenem 500 mg	Injerto piel espesor parcial
						Metronidazol 500 mg	Colgajos rotados
Eltayeb *et al*. 2016 (29)	Femenino	19	Escisión acné	No presenta	Dolor, fiebre, edema en región labial inferior, secreción purulenta	Ceftazidima 1 g/día	Desbridamiento quirúrgico
						Metronidazol 500 mg c/8 h	Drenaje quirúrgico
						Gentamicina 80 mg/día	
Kim *et al*. 2016 (30)	Masculino	61	Cirugía de implantes	Artritis reumatoide	Dolor, edema, eritema región maseterina y temporal, trismus, secreción purulenta	Ceftazidima 1 g/día	Desbridamiento quirúrgico
			Osteonecrosis	Osteoporosis		Metronidazol	Drenaje quirúrgico
				Consumo de tabaco		Vancomicina	Secuestrectomía mandíbula
						Ceftriaxona	
Ormenisan *et al*. 2015 (31)	Masculino	52	Carcinoma escamoso paladar blando	Consumo crónico de alcohol	Dolor, fiebre, disnea, edema, eritema, lesiones necróticas	Ceftriaxona	Desbridamiento quirúrgico
						Gentamicina	
						Metronidazol	Colgajo
Gelaw *et al*. 2014 (32)	Femenino	33	Inyección retrobulbar	No presenta	Dolor, edema, equimosis, eritema, disminución de agudeza visual, lesiones necróticas	Ceftriaxona	Desbridamiento quirúrgico
						Cloxacilina	
						Cloranfenicol	
						Metronidazol	

## DISCUSIÓN

La fascitis necrotizante (FN) es una enfermedad grave que rara vez se presenta en la región cervicofacial, la cual presenta un curso rápido que genera un alto riesgo de mortalidad ^11^. Con relación a las características demográficas de la enfermedad en la población estudiada, la relación hombre/mujer es de 1:1. Los factores que con mayor frecuencia gatillan la aparición de los síntomas son los traumatismos no tratados y las causas de origen odontogénico ^12^^,^^13^^-^^19^.

En la literatura, se pueden identificar factores de riesgo que promueven la aparición de FN, tales como diabetes mellitus, alcoholismo, cirrosis, arteriosclerosis, VIH, terapia con corticoides, insuficiencia renal crónica, abuso de drogas intravenosas y obesidad ^20^. De los factores de riesgo abordados en nuestro análisis, la diabetes mellitus y el consumo de alcohol fueron los más prevalentes ^11^^,^^12^^,^^21^^-^^25^. Además, la literatura menciona la presencia de tumores malignos e inmunosupresores como condiciones relacionadas ^26^^,^^27^. Sin embargo, en algunos individuos puede no existir un desencadenante evidente para el desarrollo de la enfermedad. 

El diagnóstico se determina por las características del cuadro clínico. En los estudios revisados, los signos y síntomas más frecuentes incluyen dolor, edema y eritema, junto con la presencia de tejido necrótico en el área afectada. En el caso reportado, el paciente se presentó en urgencias con un dolor intenso, edema, eritema y presencia de placas necróticas, tal como se describe en la literatura ^5^^,^^8^^,^^9^.

Al momento de diagnosticar la fascitis necrotizante (FN), es crucial considerar los signos y síntomas presentados por el paciente, así como sus comorbilidades y las causas subyacentes del cuadro clínico. Con el fin de facilitar este proceso diagnóstico, Wong *et al*. ^28^ desarrollaron un indicador de riesgo basado en pruebas de laboratorio, que ayuda a esclarecer el diagnóstico y el pronóstico del cuadro. Este indicador de riesgo se basa en seis criterios: proteína C reactiva, recuento de glóbulos blancos, hemoglobina, sodio, creatinina y glucosa, mediante los cuales los pacientes pueden ser clasificados como de bajo, moderado o alto riesgo de sufrir una infección necrotizante. No obstante, han surgido varias inquietudes sobre la fiabilidad del puntaje LRINEC debido a su limitada reproducibilidad. Por consiguiente, se sugiere complementar el diagnóstico con hemocultivos y biopsias, preferiblemente antes de iniciar la terapia antibiótica empírica ^11^^,^^12^^,^^29^.

El tratamiento quirúrgico es fundamental y siempre debe complementarse con un tratamiento antibiótico empírico, según la sensibilidad de los microorganismos involucrados. Dado que los agentes microbianos presentes pueden ser diversos, entre ellos se evidencia la presencia de estreptococos betahemolíticos del grupo A, *Klebsiella pneumoniae*, *Pseudomonas aeruginosa*, *Staphylococcus epidermis*, grupo *Streptococcus milleri*, *Acinetobacter*, *Enterobacter* y hongos ^2^^,^^3^^,^^17^^,^^30^. En nuestro caso, se identificó la presencia de Staphylococcus aureus, *Staphylococcus lugdunensis* y *Staphylococcus epidermidis*. Por lo tanto, se optó por una terapia antibiótica empírica que incluye ampicilina/sulbactam, para cubrir la microbiota anaeróbica, y cloxacilina, para combatir el estafilococo. 

A fin de garantizar un manejo eficaz de la fascitis necrotizante, es fundamental adoptar un enfoque multidisciplinario que involucre distintas especialidades médicas. Esto implica realizar un diagnóstico precoz que permita iniciar una terapia antimicrobiana de amplio espectro, llevar a cabo un desbridamiento quirúrgico rápido y realizar constantes reevaluaciones ^11^^,^^31^.

El tratamiento quirúrgico suele implicar un enfoque agresivo, con la finalidad de desbridar la totalidad del tejido afectado, lo que puede ocasionar defectos significativos en los tejidos circundantes, lo cual debe ser resuelto en función del tamaño y la localización del defecto, con el objetivo de lograr el mejor resultado funcional y estético posible. En el presente caso, el tratamiento electivo fue el desbridamiento quirúrgico, que implicó la eliminación de piel y tejido celular subcutáneo. Todos los artículos revisados respaldaron el desbridamiento quirúrgico como el tratamiento quirúrgico elegido en este tipo de casos.

## CONCLUSIÓN

La fascitis necrotizante es una enfermedad severa con un alto riesgo de mortalidad, por lo cual se debe tener especial consideración cuando aparece en lugares poco frecuentes, como la región cervicofacial. Un diagnóstico temprano basado en sus manifestaciones clínicas y estudios complementarios (imagenológicos y laboratorio) puede llevar a un correcto manejo de la patología, mediante un tratamiento oportuno, para alcanzar resultados óptimos en sobrevida del paciente, menor tiempo hospitalización, preservación funcional y estética.

## Financiamiento:

El estudio fue autofinanciado por los autores, sin ningún tipo de apoyo externo
